# Miniaturizable Chemiluminescence System for ATP Detection in Water

**DOI:** 10.3390/s24123921

**Published:** 2024-06-17

**Authors:** Giuseppe E. Capuano, Domenico Corso, Roberta Farina, Gianni Pezzotti Escobar, Giuseppe A. Screpis, Maria Anna Coniglio, Sebania Libertino

**Affiliations:** 1 Istituto per la Microeletttronica e Microsistemi—Consiglio Nazionale delle Ricerche, VIII Strada Z.I., 5, 95121 Catania, Italy; giuseppeemanuele.capuano@cnr.it (G.E.C.); roberta.farina@imm.cnr.it (R.F.); sebania.libertino@cnr.it (S.L.); 2 Department of Chemical Sciences, University of Catania, Viale Andrea Doria 6, 95125 Catania, Italy; 3 URT “LabSens of Beyond Nano” of the Department of Physical Sciences and Technologies of Matter, National Research Council (CNR-DSFTM-ME), Viale Ferdinando Stagno D’Alcontres 31, 98166 Messina, Italy; gianni.pezzottiescobar@cnr.it; 4 Department of Medical, Surgical Sciences and Advanced Technologies, University of Catania, Via S. Sofia 78, 95123 Catania, Italy

**Keywords:** ATP bioluminescence detection, silicon photomultiplier, bacterial charge detection, miniaturized sensing system, diffusion time

## Abstract

We present the design, fabrication, and testing of a low-cost, miniaturized detection system that utilizes chemiluminescence to measure the presence of adenosine triphosphate (ATP), the energy unit in biological systems, in water samples. The ATP–luciferin chemiluminescent solution was faced to a silicon photomultiplier (SiPM) for highly sensitive real-time detection. This system can detect ATP concentrations as low as 0.2 nM, with a sensitivity of 79.5 A/M. Additionally, it offers rapid response times and can measure the characteristic time required for reactant diffusion and mixing within the reaction volume, determined to be 0.3 ± 0.1 s. This corresponds to a diffusion velocity of approximately 44 ± 14 mm^2^/s.

## 1. Introduction

The presence of water on earth is essential for the development and sustenance of life, and it is largely used for anthropic activities. Water quality is an important condition for preserving human health. Many diseases can occur when water is contaminated by bacterial strains. Generally, microbial contents are measured to evaluate drinking water quality, food safety [1,2,3], and clinical analysis [4,5,6,7]. Today, more than ever, it is essential to monitor the microbiological quality of water in networks intended for human consumption to ensure public safety and health. This control not only allows for assessing the effectiveness of disinfection systems already in use in Water Safety Plans [8,9] but also for testing and developing new antibiofilm methods that can further contribute to improving water supply safety [10,11]. Several isolation and identification methods are now employed, such as conventional culturing methods, immunological protocols, molecular methods, and spectroscopic techniques. Culturing methods, also coupled with spectroscopy and mass spectrometry techniques, are usually limited to bench-top equipment requiring skilled technicians and are laborious and time-consuming. Immunological and molecular techniques for detecting bacterial pathogens are much faster than the traditional culture methods [12]. They are based on the selective interaction between a specific antibody (Ab) and a specific antigen (Ag) of the searched bacterial strain. The most used immunological techniques are the enzyme-linked immunosorbent assay (ELISA), radiometric methods (RIAs), fluorescence methods (FIAs), and chemiluminescent methods (CLIAs) [13].

However, in most cases, these methods suffer from poor specificity, sometimes returning false positive results and, then, contributing to issues of low sensitivity [14,15].

In the last decades, the polymerase chain reaction (PCR) has been considered a novel technique for bacterial strain detection. It is based on the amplification of bacterial DNA using the polymerase enzyme and consists of cycles in which DNA denaturation, primer annealing, and extension processes are involved [16,17]. Quantitative information concerning the initial amount of DNA contained in a given sample can be obtained by employing fluorescence to monitor bacterial DNA amplification in real time (“quantitative PCR” or “qPCR”) [18,19]. However, this technique requires sample preparation and skilled personnel not only in sample processing but also in interpreting the results.

Recently, researchers developed a novel molecular technique to mitigate the shortcomings of the PCR called the “isothermal technique”. The latter equally employs the nucleic-acid-based method of identification principle and works at a fixed temperature for nucleic acid amplification [20]. Amongst the different isothermal techniques employed in pathogen detection, the loop-mediated isothermal amplification (LAMP) technique has gained popularity due to its specificity, stability, and sensitivity [21,22]. Despite its widespread application, the LAMP technique encounters some limitations during usage [23]. The extraction, amplification, and identification of microbial DNA in various environmental water and food samples are quite difficult, especially when the amount of the pathogen is low. Moreover, all of these techniques require some knowledge of the DNA sequence of the specific gene that must be amplified to properly design some primers to be employed for the correct application of the methods.

It is worth knowing that the use of nanoparticles, graphene quantum dots, and electrospun nanofibers showing pathogens’ selectivity has been recently considered to improve bacterial detection techniques [24].

Currently, adenosine triphosphate (ATP)–luciferin-driven chemiluminescence is one of the most sensitive methods for bacterial charge detection [25]. ATP bioluminescent assays are reliable techniques to measure the number of viable cells [26,27,28] or biomolecules [29] in samples, widely used to indicate microbial contamination. In particular, the ATP–luciferin chemiluminescence reaction is catalyzed by the enzyme luciferase to obtain an intermediate complex (luciferyl adenylate) that combines with oxygen to produce oxyluciferin and a photon at 560 nm. The fully described reaction is as follows [30]:

(1) $$luciferin+ATP+O_{2}\underset{luciferase}{\overset{Mg^{2+}}{\to }}oxyluciferin+AMP+CO_{2}\;+pyrophosphate+h\nu$$

Santangelo et al. [31] developed the prototype of a low-cost, miniaturized, and highly sensitive ATP detection device employing the ATP–luciferin chemiluminescent reaction and based on 3D printed lab-on-chip microfluidics (LoC) [32,33,34] coupled with a non-commercial 0.4 × 0.4 mm^2^ silicon photomultiplier (SiPM, fabricated by STMicroelectronics) for highly sensitive, continuous, and real-time ATP detection, reaching a limit of detection (LoD) equal to 8 nM and sensitivity of 1.82 × 10^−2^ A/M; the authors also performed static measurements by replacing the 3D chip with a glass cuvette, reaching a LoD equal to 3.7 nM and a sensitivity of 9.32 × 10^−2^ A/M (ampere/moles). The SiPM is a solid-state photodetector consisting of a bi-dimensional array of microcells (pixels), each one composed of a series of quenching resistors and a single photon avalanche diode (SPAD), operating in Geiger mode [35,36,37,38]. It is characterized by high quantum efficiency, high gain, high speed, low operating voltage, simple output electronics, and single photon sensitivity, useful for the detection of very low photon fluxes.

Hu et al. [39] developed a low-cost and portable device based on the ATP–luciferin-driven chemiluminescence reaction taking place in test tubes for bacterial charge detection, where the main components of the acquisition system are a low-noise photoelectric signal detection and a processing circuit based on an SiPM, a power management module, and a high-performance embedded microcontroller subsystem with peripheral circuits. The authors adopted the balanced chopper modulation and lock-in amplification techniques to improve the signal-to-noise ratio and performed a zero-adjustment to eliminate the dark current of the SiPM and increase the dynamic range of the system, detecting a minimum ATP concentration of 3.6 × 10^−11^ M and obtaining a sensitivity of 0.387 mV/M. However, the proposed system requires complex electronic circuitry and the material to be tested must be sampled and inserted in the instrument by a human operator.

Some commercial devices developed for cellular ATP detection reach low LoD. For example, the BioThema ATP Kit SL [40] allows the monitoring of the ATP concentration ranging from 10^−12^ to 10^−6^ M; water samples are treated and inserted into cuvettes or microplates along with the relative reactants, and the measurements are performed through an appropriate luminometer. The Luminultra Quench-Gone Aqueous (QGA) Test Kit [41] is optimized to measure down to 0.1 ng/L (which corresponds to about 2 × 10^−13^ M of ATP, considering its molecular weight A_ATP_ = 507.181 g/mol [42]). In this case, the ATP sample is extracted through filtering, diluted, and inserted into microtubes employed for measurement with a luminometer. The Hygiena handy type luminometer [43] allows the monitoring of the bacterial charge in surfaces to be sanitized, returning information about the cleaning status. In particular, each sample is obtained by using a swab, where the latter is treated and inserted into the luminometer; the surface is considered clean if the system returns concentration values below 20 relative light units (RLU). For all systems, the operator has to prepare the sample to be measured (either by filtering and mixing water samples with reactants or by swabbing a surface and dipping the swab into buffer solutions). It means they depend on the operator’s expertise and the measurement requires a long time for sample preparation.

In this work we propose a low-cost and very high-sensitivity bacterial detection system based on the ATP–luciferin-driven chemiluminescence reaction taking place in a static regime, aiming at reaching a low LoD and a high sensitivity. The system is mainly composed of a 5 × 5 mm^2^ base-squared quartz cuvette with five optical windows (the lateral ones are appropriately covered with aluminum to avoid the dispersion of the emitted photons) where the ATP–luciferin-driven reaction takes place, and of a 6.07 × 6.07 mm^2^ commercial SiPM posed under the quartz cuvette, which detects the photons emitted during the reaction. The output signal is a current proportional to the intensity of the emitted light and, consequently, to the ATP concentration. The device possesses simple electronic circuitry and does not need the presence of human operators for sampling and sample treatment, allowing it to perform in situ, remote-sensing, and real-time measurements.

## 2. Materials and Methods

### 2.1. SiPM Details

The SiPM utilized in this study is a commercial device manufactured by OnSemiconductor [44] (Phoenix, AZ, USA, MICROFJ-60035-TSV-TR1). It possesses dimensions of 6.07 × 6.07 mm^2^, housing a total of 22,292 pixels, each consisting of a Single Photon Avalanche Diode (SPAD) operating in Geiger mode [38] and linked to a quenching resistor. The SiPM exhibits a measured breakdown voltage of −24.5 V [44], falling within the voltage range specified by the manufacturer (reverse voltage of 24.2 V–24.7 V).

The operating voltage (V_op_) was determined by considering an overvoltage of −2.5 V, chosen to achieve a high gain (2.9 × 10^−6^ in this instance). For the measurements conducted in this study, the SiPM was biased at Vop = −27 V. At this operating voltage, the detector exhibits a Dark Count (DC) of 5 × 10^4^ Hz/mm^2^, corresponding to a dark current of 9.0 × 10^−7^ A, with an 8% crosstalk probability [44]. The measured dark current of 1.06 × 10^−6^ A aligns closely with the value provided in the datasheet.

**Figure 1:**
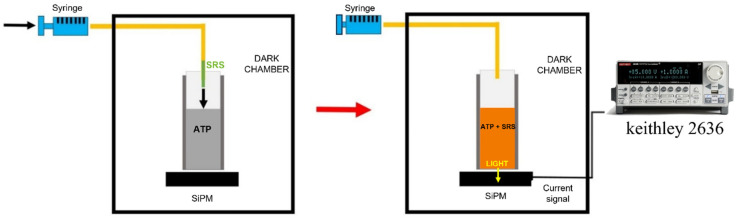
The schematic layout of the ATP detection system in a static configuration.

Measurements were carried out by biasing the SiPM using a Source Meter Multi-Channel I-V, KEITHLEY 2636 (Tektronix, Beaverton, OR, USA 2600 Series SourceMeter^®^), employing a Lab-VIEW^®^ (National Instruments, Austin, TX, USA, Version 2021) routine to control the bias voltage and measurement parameters (such as time and integration time), to acquire the current signal, and to automatically record data. Subsequently, the collected data were analyzed offline using a Matlab^®^ (Mathworks, Natick, MA, USA, Version 2023) routine (see Figure 1 for a schematic of the measurement setup).

### 2.2. Setup Description

The proposed detection system is formed by the SiPM, already described in Section 2.1, and a 5 × 5 mm^2^ base-squared quartz cuvette with 5 optical windows coupled together (Hellma, Müllheim, Germany, Micro-cuvette 111-057-40 QS). The cuvette sides were appropriately covered with aluminum to collect all the produced light, hence maximizing the electric signal related to the chemiluminescent reaction. The cuvette was filled with 100 μL ATP (see after for preparation details) at different concentrations; then, the standard reaction solution (SRS) containing the luciferin was added through a hand-made pumping system, composed of a syringe linked to a silicon tube. The former was positioned out of the dark chamber containing the detection system (not reached by the environmental light), while the silicon tube was long enough to enter the chamber through a light-shielded entrance. A schematic representation of the experimental set-up is shown in Figure 1. The syringe–tube system delivered 100 μL SRS into the cuvette. In this way, not only can the mixing and the reaction take place while the chamber is closed, but also the syringe–tube device allows one to pump the SRS into the cuvette after the data acquisition is started, obtaining detailed information on the characteristic reaction time required by the reactants to mix in the reaction volume (200 μL), since the reaction occurs instantaneously at room temperature [45].

### 2.3. Standard Preparation and ATP Bioluminescence Measurements

ATP Determination Kit (A22066) from Molecular Probes [46] was used as a reference for the quantitative determination of ATP concentration with recombinant firefly luciferase and its substrate D-luciferin. It is based on the reaction reported in Equation (1). The assay involves the enzyme luciferase to catalyze luciferin oxidation, where magnesium ions (Mg^2+^) are considered enzyme cofactors. The ATP concentration is proportional to the amount of emitted light (560 nm) released along with carbon dioxide (CO_2_), adenosine monophosphate (AMP), and pyrophosphate. The SRS was prepared using MilliQ water from Simplicity UV (Millipore, by Merk, Darmstadt, Germany), 20× Reaction Buffer, dithiothreitol (DTT) to a concentration of 0.1 M, D-luciferin to a concentration of 10 mM, and firefly luciferase (5 mg/mL). The ATP concentrations considered for the chemiluminescence measurements were 500 nM, 250 nM, 125 nM, 62.5 nM, 31.25 nM, 15.62 nM, 7.81 nM, 3.90 nM, 1.95 nM, 0.98 nM, 0.50 nM, and 0.25 nM, starting from the concentration of 5 mM provided by the ATP Determination Kit. Finally, three sets of ATP concentration measurements were taken between 0.25 nM and 500 nM to obtain a satisfying statistic at each concentration.

## 3. Results

**Figure 2:**
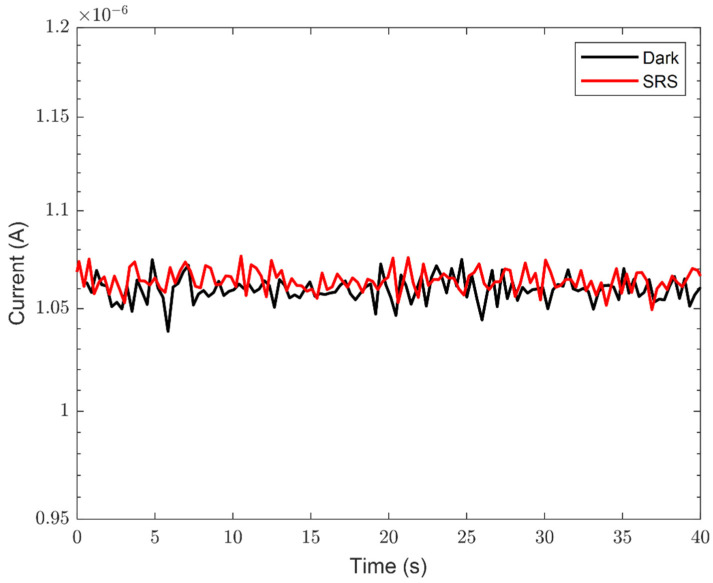
The current versus time measurements of the dark current (black curve) and the signal obtained by using only SRS (red curve).

Before proceeding with the ATP detection measurements, the system’s dark conditions and the absence of optical sources interfering with the measurements were verified. Figure 2 reports the comparison between the dark count noise signal and the one obtained by filling the cuvette with only SRS to highlight the absence of optical interferents during the measurements. Therefore, the static performances of the ATP detection system were tested by employing different ATP concentrations ranging between 0.25 nM and 500 nM (see Section 2 for details).

**Figure 3:**
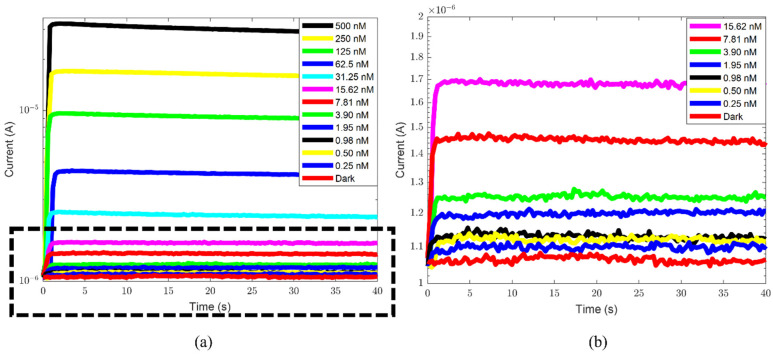
(**a**) The current signal versus time as a function of ATP concentration, for one of three sets of measurements. (**b**) The zoom-in of the region identified by the black dashed rectangle shown in (**a**). The bottom red curve is the dark current signal. The legend refers to the measured ATP concentrations, with values ranging from 0.25 nM to 500 nM (upper black line 500 nM, upper yellow line 250 nM, upper green line 125 nM, upper blue line 62.5, cyan line 31.25 nM, magenta line 15.62 nM, red line 7.81 nM, green line 3.90 nM. blue line 1.95 nM, black line 0.98 nM, yellow line 0.50 nM, and blue line 0.25 nM).

Figure 3a summarizes one of three sets of current measurements in the static configuration as a function of time for the different ATP concentrations. The data show an increase in the optical signal as the ATP concentration in the sample increases. Figure 3b shows a zoom-in of the measurements at low concentrations and allows the verification that even at the lowest measured value the signal is above the dark current. The signal is quite stable, decreasing slowly with time, as already observed in ref. [31]. In particular, the current signal reduces as a function of time more rapidly at higher ATP concentrations.

**Figure 4:**
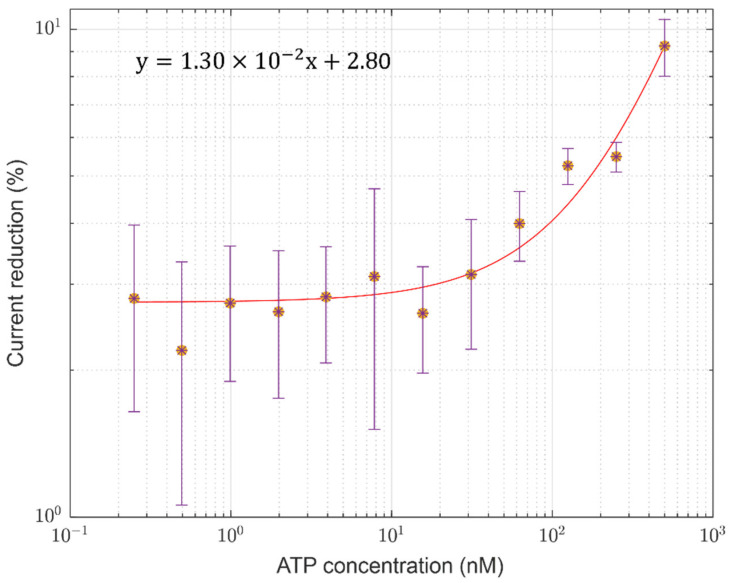
The current reduction percentage calculated in the time range from 15 s to 35 s, as a function of ATP concentration. Each value (*) and its error bar have been obtained by considering the average and the standard deviation over three measurements taken at each ATP concentration, along with the noise associated with each measure. A linear fit has been performed (solid red line); “x” and “y” in the equation shown in the upper left of the figure represent the ATP concentration and the fit values, respectively.

To study the current reduction as a function of time, the range from 15 s to 35 s was defined. The ratio between the current values at 15 s and 35 s was calculated for each ATP concentration and considering all three sets of measurements. The data, expressed as percentages, are summarized in Figure 4.

This range has been chosen to guarantee that the current is reducing. In fact, for the first few seconds, the signal is stationary, while for longer times the reduction rate changes, going asymptotically toward the dark current. The signal decreases similarly for concentrations lower than 15.62 nM, with a reduction of about 3%. For concentrations higher than 15.62 nM, the current reduction percentage increases with ATP concentration, following the trend shown in Figure 4. At 500 nM, the current reduction is 9% of the starting value, about three times higher than the reduction measured at low ATP concentrations. It is worth noting that current reduction values have been estimated by performing polynomial fits over each current curve in a decreasing phase relative to each set of measurements for each ATP concentration (such as those reported in Figure 3). The observed trend can be explained by considering a faster consumption of the SRS reactant at higher ATP concentrations.

**Figure 5:**
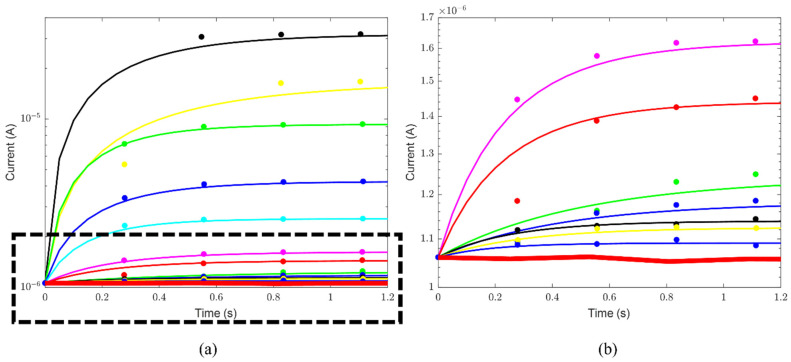
(**a**) The current signal as a function of time, with the time axis ranging between 0 s and 1.2 s, for different ATP concentrations. (**b**) The zoom-in of the region identified by the black dashed rectangle shown in (**a**) is reported. The bottom red curve in (**a**,**b**) indicates the dark noise signal. The fits performed on each curve by following Equation (2) are also reported as solid lines. ATP concentrations range from 0.25 nM to 500 nM. The set of measurements shown in Figure 3 is considered. To identify the curve color–ATP concentration relation, refer to the legends shown in Figure 3.

To correctly analyze the data, a study was performed on the first few seconds from the SRS injection. In fact, after inserting the SRS into the cuvette containing the ATP solution, the current signal *I*(*t*) increases from the reference (dark noise) to the maximum current value *I*_max_ corresponding to each ATP concentration by following the trend reported in Equation (2):

(2) $$I\left(t\right)=I_{\max\;}\left(1-e^{-\frac{t}{\tau }}\;\right)$$

where *t* is the time variable, and *τ* is the characteristic increasing time in correspondence to which the current intensity is equal to $(1-1/e)\cdot I_{\max\;}\approx \;0.63\;I_{\max\;}$(see Figure 5).

**Figure 6:**
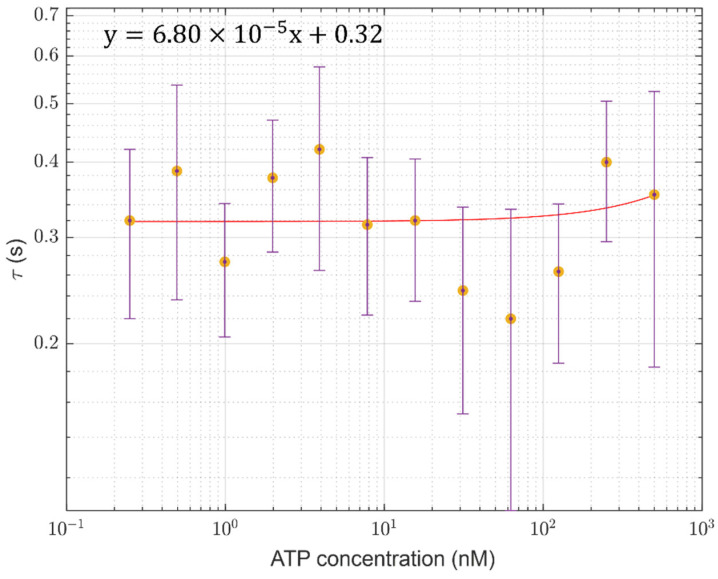
Values of the estimated characteristic SRS diffusion time (τ) as a function of ATP concentration. Each value (*) and its error bar have been obtained by considering the average and the standard deviation over three measurements performed at each ATP concentration. The fit performed over the points (solid red curve) is also shown, suggesting a constant trend of the diffusion time with the concentration. The terms “x” and “y” in the equation shown in the upper left of the figure represent the ATP concentration and the fit values, respectively.

The data analysis of all three sets of measurements demonstrates that *τ* is almost the same for all of the concentrations, obtaining a value of 0.3 ± 0.1 s (see Figure 6). It is strictly related to the diffusive mechanism characterizing the SRS mixing with ATP solution into the reaction volume. If the diffusion of the SRS reactant starts from a single point toward the surrounding space into the reaction volume under a concentration gradient, and approximating the reaction volume *V* to a sphere with radius $r=\sqrt[{3}]{3V/4\pi }=3.6\;mm$, the SRS diffusion velocity *D* can be estimated using the following expression:

(3) $$D=\frac{r^{2}}{\tau }\approx 44\pm 14\;mm^{2}s^{-1}$$

If the firefly luciferase molecule drives the diffusion mechanism (considering its remarkable weight, i.e., 62 kDa [47]), it is possible to verify the relationship between the experimental diffusion velocity and the firefly luciferase dimensions in a single-point diffusion scenario. If the presence of turbulent mechanisms is neglected, the energy dissipated through the viscous friction force of the diffusing molecules can be equaled to their thermal energy, obtaining the following:

(4) $$6\pi \eta RD=K_{B}T$$

where *K_B_* is the Boltzmann constant, *T* = 293 K is the temperature at which the diffusion took place, *η* is the solution viscosity (assumed equal to the water viscosity, that is, 1.0016 × 10^−3^ Pa s at 20 °C [48]), and *R* is the radius of the diffusing molecule (approximated to a sphere). Considering the molecular weight *M* of the firefly luciferase, which is 62 kDa, the radius of the diffusing molecule can be estimated by considering the following expressions (reported in Equations (2.1) and (2.2) of ref. [49]):

(5) $$V_{M}=\frac{0.73\;cm^{3}g^{-1}\times 10^{21}\;nm^{3}cm^{-3}}{6.023\times 10^{23}\;Da/g}\times M\;\left(Da\right)=1.212\times 10^{-3}\times M$$

(6) $$R=\sqrt[{3}]{3V_{M}/4\pi \;}=0.066\times \sqrt[{3}]{M}=2.6\;nm$$

where *V_M_* is the volume of the molecule and 0.73 cm^3^/g is the reciprocal of the mean density of all proteins (about 1.37 g/cm^3^). The expected diffusion velocity can be inferred through the following expression (also referred to as the “Stokes–Einstein” equation) [50]:

(7) $$D=\frac{K_{B}T}{6\pi \eta R}$$

obtaining a velocity of 8.3 × 10^−5^ mm^2^/s; this value is five orders of magnitudes lower than that estimated experimentally, implying a diffusion time of 1.6 × 10^5^ s (about 44 h).

The large difference between the expected and measured diffusion velocity values is due to the assumptions considered in the model used. The reaction volume is not spherical (as assumed previously) and the mixing of the SRS with the ATP solution is helped by the direct SRS injection into the cuvette (with a starting velocity different from zero), which acquires a not-negligible kinetic energy before the mixing, generating turbulences and then facilitating its diffusion into the ATP solution. This process remarkably reduces the diffusion times concerning those associated with the condition of single-point diffusion assumed in the model. By dividing the radius associated with the reaction volume by the experimental diffusion time, the mean velocity along one direction can be estimated as follows:

(8) $$v=\frac{r}{\tau }=12\pm 4\frac{mm}{s}$$

which is a reasonable value for the SRS velocity during the injection process.

**Figure 7:**
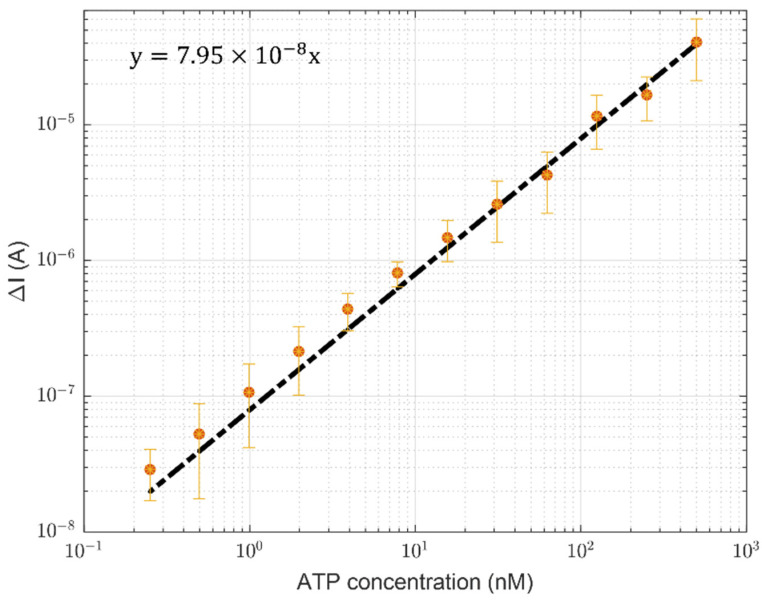
The calibration curve of the detection system described in this work. The vertical axis reports the difference between the measured maximum current signal and the dark noise (ΔI). Considering all three sets of measurements, the mean maximum current value and the respective error bar for each concentration have been estimated.

For each set of measurements, we collected the maximum current *I_max_* as the average current acquired in a time range going from 2 to 5 s. Following this procedure, we are confident the solution is already in the “fully mixed” regime, before the current reduction range, and the time interval used to average the signal is enough to have a reasonable number of measurement points. For each curve, the maximum current value has been associated with the relative ATP concentration. The minimum value measured with the detection system is 0.25 nM (see Figure 7), more than an order of magnitude lower than the LoD inferred by Santangelo et al. [31] in the static regime (LoD = 3.7 nM) and about an order of magnitude higher than the minimum ATP concentration detected by Hu et al. [38] (3.6 × 10^−2^ nM). In particular, the LoD value inferred with the system proposed in this work is 0.2 nM, estimated by using the following equation [51]:

(9) $$LoD=3\times \frac{SD}{slope}$$

where *SD* is the standard deviation of the reference signal, and the *slope* (sensitivity) refers to the linear fit of the calibration curve (that associates each maximum current value with the corresponding ATP concentration). In this case, the measured *SD* is equal to 5.5 × 10^−9^ A, while the sensitivity is 79.5 A/M, much higher than the values reported in the literature [31,39]. The sensitivity is also higher than typical sensitivities of high-cost and bulky bench-scale commercial laboratory luminescence devices [40,41,43], although these show very low LoD thanks to the preliminary application of filtering processes.

By knowing the mean amount of ATP characterizing a living cell, the bacterial concentration in a water sample can be inferred by detecting the ATP extracted from bacterial cells through appropriate procedures. For this purpose, it is possible to define the Microbial Equivalent (ME) unit [52], where it is assumed that each bacterial cell holds 1 fg of ATP, which is the average amount for a typical *Escherichia coli* cell. Therefore, by knowing the ATP molecular weight A_ATP_ = 507.181 g/mol [42], a single living cell contains a mean ATP amount of 2 × 10^−18^ mol. This means that the minimum ATP concentration detectable with the proposed system (LoD = 0.2 nM) corresponds to a minimum detectable bacterial concentration of 10^8^ cells/L.

For bacterial load determination in water samples, pretreatment is carried out in commercial applications [40,41,43]. The water sample is filtered, and the filter (containing the bacteria) is attached to a syringe barrel to start the lysis process. This is achieved by adding 1 mL of lysis buffer to the barrel; it allows the bacterial degradation for both gram-negative and gram-positive bacteria to pass gently through the filter as a consequence of lysozyme activity toward the cell wall. This step is swift; hence, to increase sensitivity, often additional samples are filtered and the lysis process is repeated. Once all bacteria have been disrupted, soluble proteins and nucleic acids are released and ATP is dissolved in a tube containing an ATP dilution reagent and ready to be tested. For our application, we aim to avoid the sample concentration. The final goal of our approach is to avoid, or at least reduce, the amount of water to be filtered.

## 4. Conclusions

In this work, we describe the design, fabrication, and testing of a low-cost and miniaturized chemiluminescent sensing system for highly sensitive real-time ATP detection. The system is mainly composed of a five-optical-windows quartz squared-based cuvette with the sides appropriately covered with aluminum, coupled with an SiPM as a photodetector. The process of optical cuvette wrapping allows the collection of all the light produced during the reaction, while the use of an SiPM provides a high-quality output signal compared to traditional systems due to its enhanced characteristics. The limit of detection of the proposed system is 0.2 nM, while the sensitivity is equal to 79.5 A/M, much higher than the typical sensitivities of bench-scale commercial laboratory luminescence devices. Moreover, the developed system allows one to obtain a direct measurement of the characteristic time required by the reactants to diffuse and mix after injection in the reaction volume (200 μL), equal to 0.3 ± 0.1 s, through which a diffusion velocity of about 44 ± 14 mm^2^/s has been inferred. Commercial bench devices require the presence of human operators and longer times to perform the same measurements as the proposed system. It can be implemented in situ to monitor water’s total bacterial charge due to its capacity to perform highly sensitive, in situ, remote-sensing, and real-time measurements, with a minimum detectable total bacteria concentration of about 10^8^ cells/L. Future work that has to be considered of primary importance is the development of early warning systems, thanks to the device characteristics such as miniaturization, portability, and low cost, making it ideal for a network of integrated sensors in the surrounding environment to obtain a qualitative pre-screening of water.

## Data Availability

The raw data supporting the conclusions of this article are available in the figures. Ascii files will be made available by the authors upon request.
